# Epidemiological features and pathological study of avian leukosis in turkeys’ flocks

**DOI:** 10.14202/vetworld.2017.1135-1138

**Published:** 2017-09-26

**Authors:** Mourad Zeghdoudi, Leila Aoun, Latifa Merdaci, Nardjes Bouzidi

**Affiliations:** 1 Department of Veterinary Medicine, Chadli Bendjedid University, El Tarf-36000, Algeria; 2 Department of Biology, Chadli Bendjedid University, El Tarf-36000, Algeria

**Keywords:** enzyme-linked immunosorbent assay, epidemiological features, histopathology, lesions, leukosis, turkeys

## Abstract

**Aim:**

The purpose of this study was focused on the identification of tumor diseases in turkeys on the basis of a detailed description of epidemiological features, clinical signs, lesions, and histopathological changes.

**Materials and Methods:**

Outbreak of a tumor disease in turkeys was investigated in various regions of Eastern Algeria. Four turkeys’ flocks aged from 17 weeks were affected, resulting to mortality often over 10%, on a period of 15 days. The main epidemiological characters, clinical signs, and lesions were observed throughout all the course of the disease. Serum samples were collected from affected turkeys in each flock to detect p27 antigen in enzyme-linked immunosorbent assay (ELISA) test to diagnose avian leukosis virus (ALV). Portions of sciatic nerves and livers are taken from dead turkeys for microscopic examination.

**Results:**

The disease was characterized by clinical signs such as anorexia, weakness, and diarrhea. Necropsy of the dead birds showed hepatomegaly and gross splenomegaly with neoplastic nodules or gray foci and diffuse infiltration in the myocardium and lungs. ALV antigen test using ELISA confirmed the presence of virus leukosis. Histopathological sections of the liver had proliferations of lymphoblastoid cells and absence of any modifications or lymphocytic infiltration in peripheral nerves.

**Conclusion:**

The present study confirms that this disease condition is caused by lymphoid leukosis.

## Introduction

Tumor diseases represent a great challenge for biomedical and veterinary scientists so that to understand the real causes of neoplasia in humans and animals. Although the research on avian leukosis virus (ALV) has lasted for more than a century, there are no vaccines yet to protect birds from this infection [1]. ALVs are prevalent throughout the world, and new strains which arise in some regions may spread over boarding areas, thereby undermining national disease control measures [2]. In some countries, leukosis has now been eradicated from commercial broiler breeder flocks by the rigorous application of eradication programs, but in other countries, the disease remains and even has spread to layer flocks. ALV has been eradicated in the United States from the pure lines and the parents, which have greatly reduced the incidence of infection in both layer flocks and broilers [3].

Tumor diseases referenced in birds are the *Reticuloendotheliosis virus* (REV) [4], lymphoproliferative disease (LPD) [5], and lymphoid leukosis (LL) [4] whose common etiologic agent is a retrovirus. Pennycott and Venugopal [6] reported that Marek’s disease (MD) induced by herpesvirus can be expressed in turkeys by a tumor development. Tumor diseases are few in commercial poultry, and the reports of clinical diseases in turkeys are rare. Some cases have been described from the USA and England; however, in Algeria, tumor diseases in turkeys were not reported until now. Disease manifestations which can arise by viral mutation [7] present similarities and difficulties to identify this disease by virological or biological studies that do not establish a diagnosis unequivocally [8]. Because neoplastic viruses are widespread among chickens, virus isolation and the demonstration of antigen or antibody have limits in diagnosing cases of lymphomas.

The purpose of the study is to identify the tumor diseases in turkeys associated with clinical signs, lesions, histological changes, and the main epidemiological features which can complete the findings in the latest works.

## Materials and Methods

### Ethical approval

This study does not require the approval of the Institute Animal Ethics Committee.

### Study area and animals

During the year 2015, we visited four affected flocks of commercial rearing turkeys, in the areas of Skikda, Guelma, Annaba, and El Tarf, in the East of Algeria. The respective flocks of 1500, 2000, 1200, and 1500 birds aged 1 day were imported from Italy and from the same hatchery. These flocks were vaccinated according to the official vaccination plan (RTI vaccine against infectious rhinotracheitis, Dindoral vaccine against necrotic enteritis, and Hitchner B1 and La sota against Newcastle disease). Parents of respective turkey flocks were vaccinated against MD. Until the age of 4 months, the livestock appeared to grow up normally, and mortality rates did not exceed 5%. The occurrence of high mortality began from the 4^th^ month with an average of 20 dead birds per day.

### Serological diagnosis

The commercial enzyme-linked immunosorbent assay (ELISA) test kit remains the first choice for the identification of ALV in the poultry industry. Using a commercial ELISA kit (IDEXX Laboratories, Westbrook, ME, USA), according to the manufacturer’s instructions, serological study was performed by double antibody capture sandwich ELISA which can detect the presence of p27 antigen common to all subgroups of ALV. The rate of p27 was measured photometrically at 405-410 nm to estimate the level of ALV infection. Blood samples were collected from sick turkeys in each flock and sent to the laboratory of epidemiology research of Chadli Bendjedid University (ESSPRETCADS). Serum was separated from the blood and stored frozen at −20° until tested. The serum samples were tested in duplicate to ensure the accuracy of results. The seropositive flocks were considered to be infected with ALV when the Elisa units (EUs) were >65 EUs.

### Necropsy and histological study

Postmortem examinations during the course of the disease outbreak were carried out on dead birds of >24 h. Livers and sciatic nerves have been forwarded to the laboratory of histology in the veterinary department of El Tarf University for histopathological examination. Portions of sciatic nerves were fixed in 10% formol sublimate, and histological sections were stained with hemalun eosin safran. Sections of liver were fixed in 10% buffered formol saline, embedded in paraffin wax, and were routinely stained with hematoxylin and eosin for microscopic examination. MD and retroviruses were diagnosed on the basis of clinical signs, gross lesions, and histopathology [5].

Differential diagnosis has to depend on an examination of stained sections of peripheral nerves and the livers to determine the histological and cytological characteristics of the lesions. In a small minority of cases, a decision cannot be made even when a detailed microscopical examination is possible.

## Results

The most consistent epizootiological features showed that the disease occurred regardless of the season, between 17 and 19 weeks of age with an average mortality rate of 10, 9%, during a period of approximately 14 days (Table-1).

**Table-1:** Summary of epidemiological features.

Flocks	Skikda	Annaba	El Tarf	Guelma
Number of turkeys	1500	1200	1500	2000
Age (weeks)	18	18	19	17
Mortality until 4 months	5.2%	5.7%	5.5%	4.9%
Period of disease appearance	July	July	January	February
Duration of clinical disease (days)	15	16	12	15
Mortality during the disease	12.5%	11.2%	9.6%	10.3%

**Figure-1:**
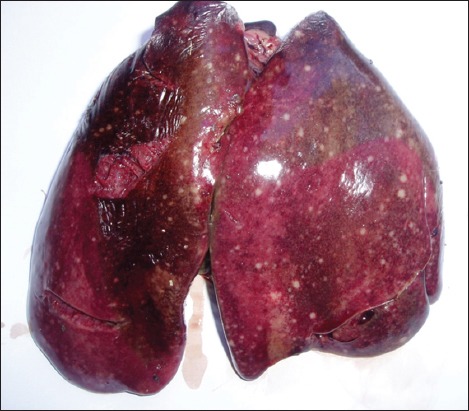
Enlarged liver with scattered small nodules.

**Figure-2:**
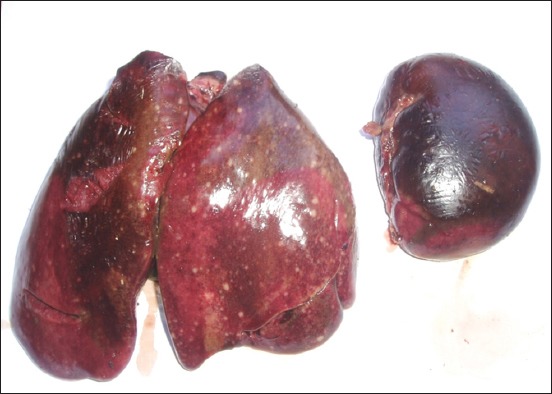
Spleen greatly enlarged up to 5 times normal size.

**Figure-3:**
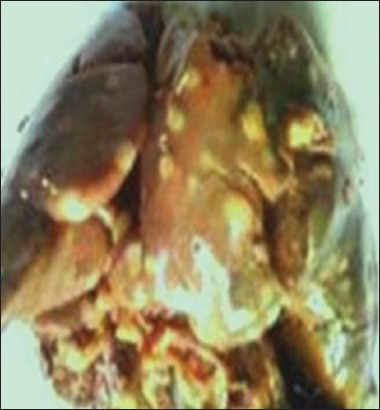
Whitish liver containing nodules of different sizes.

**Figure-4:**
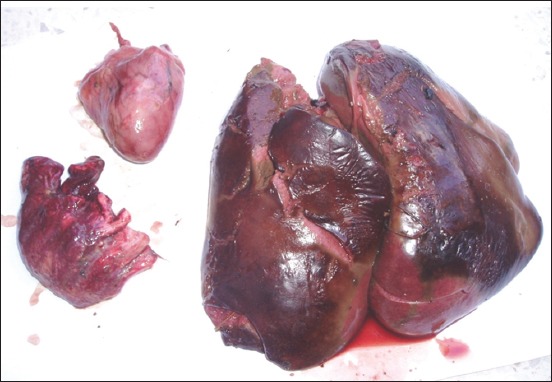
Torn liver due to hypertrophy, lung pale distorted by the presence of tumors and nodules in the myocardium.

The clinical signs and lesions were similar in the four sites. Symptoms observed were weariness, anorexia, nonspecific diarrhea, and death in 24-48 h. Necropsy performed on 253 bodies showed that all the lesions have been localized in the liver, spleen, heart, and lungs. The liver was diffusely enlarged containing small or miliary nodules <1 mm in diameter (Figure-1), as well as the spleen was greatly enlarged up to 5 times normal size (Figure-2). In addition to the miliary form which is most obvious, tumors of different sizes were present throughout the liver in some birds. Tumors are soft, smooth, and glistening varying from 2 to 5 mm in diameter and may occur in large numbers (Figure-3). Diffuse tumoral lesions were apparent in the myocardium and the lungs (Figure-4).

**Figure-5:**
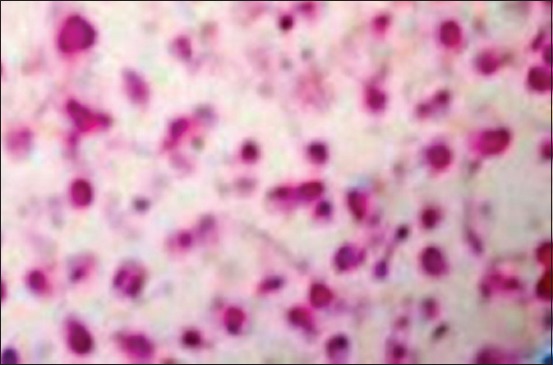
Proliferation of lymphoblastoid cells and mitotic figures (*400).

**Figure-6:**
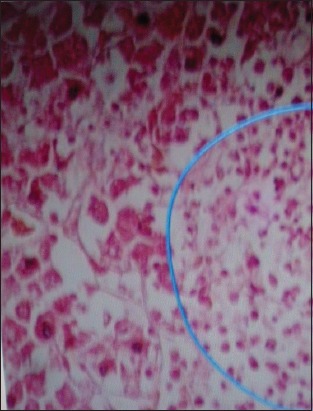
The normal tissues of the liver parenchyma were replaced by lymphoblastoid cells (*200).

The serological test showed that all serum samples were positive to ALV in ELISA with values >75 EUs. Examination by microscopy (*400) showed no presence of lymphocytes in the structure of nerves. However, histological liver sections revealed a uniform population of lymphoid cells. These cells appeared to be predominantly lymphoblastoid in type, and mitotic figures were easily identifiable (Figure-5). Neoplastic cells have replaced the normal tissues of the liver parenchyma (Figure-6).

## Discussion

LL is the most feared disease of poultry. Reports of clinical disease in poultry are few and totally rare for turkeys. In Algeria, it is the first case of turkey’s tumor disease, and the present study was conducted to provide an update on the development of the disease.

Vaccination of turkey breeders against MD ensures health protection to their progeny, which moves us away from the suspicion of MD. The appearance of the disease only in turkeys, which in addition have the same origin, suggests a vertical transmission of the virus. Extensive studies have confirmed vertical transmission of LL from one generation to another [4]. There are, however, no reports of vertical transmission of REV from infected turkey breeder flock.

The present disease was manifested by clinical signs from the 17^th^ week and an average mortality rate of 11% over a period of 15 days before the reform. Because the incubation period is rarely <14 weeks, LL is usually a neoplastic disease of breeders and commercial egg-layers but not of broilers [9]. The previous reports on leukosis indicated the sporadic nature of this disease with low levels of mortality [5].

RE and LPD are differentiated by a spectacular enlargement of the liver and spleen in the case of REV with nodular monomorphic lesions while the LPD is manifested without signs, with a moderate hypertrophy and an infiltration of polymorphic aspect [10]. Biggs *et al*. [4] described the LDP by the infiltration of peripheral nerves and splenomegaly, and also, Okoye *et al*. [11] reported in REV infection and infiltration of peripheral nerves. McDougall *et al*. [12] reported that LL is manifested by a liver 3-4 times larger than normal, compatible with the lesions observed in this study and a rate less enlarged, in contradiction to what we found. In birds suspected for LL, the liver was soft, friable, and grayish white and showed diffuse enlargement [13].

Histological study showed that the uniform nature of the lymphoblasts remained obvious and nearly all lymphoblasts undergo karyorrhexis simultaneously and mitotic figures. Mays *et al*. [14] reported cases of LL in turkeys characterized by a proliferation of lymphoblastoid cells, a mortality rate of 14%, and a similar trend to that of our study. The clinical study and histopathological lesions of the present study indicated that neither MD nor LPD and REV were in relation to what we have described.

## Conclusion

This study shows that this disease is caused by LL which unfortunately still exists among avian rearing. The identification of this communicable disease is a fatality for avian industries and would lead to considering radical solutions and extreme conditions of biosecurity. However, this study suggests that research should be oriented toward epidemiological characteristics, the understanding of which would allow us to hypothesize the relationship between this disease and growth hormones**.**

## Authors’ Contributions

MZ conceived and designed the study. LA performed the study. LM analyzed the data. NB wrote the paper. All authors read and approved the final manuscript.
